# DNA Methylation Sustains “Inflamed” Memory of Peripheral Immune Cells Aggravating Kidney Inflammatory Response in Chronic Kidney Disease

**DOI:** 10.3389/fphys.2021.637480

**Published:** 2021-03-02

**Authors:** Xiao-Jun Chen, Hong Zhang, Fei Yang, Yu Liu, Guochun Chen

**Affiliations:** ^1^Department of Nephrology, The Second Xiangya Hospital, Central South University, Changsha, China; ^2^Hunan Key Laboratory of Kidney Disease and Blood Purification, Changsha, China; ^3^Department of Cardiovascular Surgery, The Second Xiangya Hospital of Central South University, Changsha, China

**Keywords:** chronic kidney disease, DNA methylation, inflammation, peripheral immune cells, epigenetic memory

## Abstract

The incidence of chronic kidney disease (CKD) has rapidly increased in the past decades. A progressive loss of kidney function characterizes a part of CKD even with intensive supportive treatment. Irrespective of its etiology, CKD progression is generally accompanied with the development of chronic kidney inflammation that is pathologically featured by the low-grade but chronic activation of recruited immune cells. Cumulative evidence support that aberrant DNA methylation pattern of diverse peripheral immune cells, including T cells and monocytes, is closely associated with CKD development in many chronic disease settings. The change of DNA methylation profile can sustain for a long time and affect the future genes expression in the circulating immune cells even after they migrate from the circulation into the involved kidney. It is of clinical interest to reveal the underlying mechanism of how altered DNA methylation regulates the intensity and the time length of the inflammatory response in the recruited effector cells. We and others recently demonstrated that altered DNA methylation occurs in peripheral immune cells and profoundly contributes to CKD development in systemic chronic diseases, such as diabetes and hypertension. This review will summarize the current findings about the influence of aberrant DNA methylation on circulating immune cells and how it potentially determines the outcome of CKD.

## Introduction

Over the past decades, the incidence of chronic kidney disease (CKD) has rapidly increased worldwide (GBD Chronic Kidney Disease Collaboration, 2020), likely due to the huge changes in human living habits and the environment. A subset of CKD is characterized by a gradual loss of kidney function over time even with intensive supportive treatment and thereby irreversibly progresses to end-stage renal disease (ESRD). Epidemiological studies have revealed that all stages of CKD are correlated with greater risks of cardiovascular morbidity, premature death rates, declined quality of life, and tremendous economic burden (Cockwell and Fisher, 2020). In 2017, the number of deaths caused by CKD reached 1.2 million, known as the 12th leading causes of global death (DALYs GBD and Collaborators H, 2018). Undoubtedly, CKD is one of the biggest threats to global health as well as one of the top challenges to limited medical resources in most countries. Because multiple factors contribute to the disease progression, current therapeutic strategies to manage CKD mostly rely on the control of the detectable abnormalities, like proteinuria, hyperglycemia, hypertension, and so on. However, a proportion of CKD still progresses to ESRD even when these mentioned disadvantages are fully under control. For example, compelling evidence from multiple large-scale clinical trials remains insufficient to definitively conclude a relative risk reduction by intensive glycemic control for long-term diabetic kidney disease (DKD) exposures, which are generally accompanied by chronic hyperglycemia (Hemmingsen et al., 2011). A more in-depth understanding of the underlying molecular mechanisms implicated in the pathogenesis of CKD remains necessary for the development of novel therapeutic strategies.

Chronic kidney inflammation in the process of CKD is featured by the diffusive interstitial infiltration of various immunocytes, including T lymphocytes, B lymphocytes, neutrophils, and monocytes. In general, the function of leukocytes trafficking to the kidney is to eliminate pathogens, remove necrotic cells and tissue debris from the original insult, and finally facilitate kidney tissue repair. The infiltrated leukocytes produce abundant cytokines and growth factors to establish an inflammatory milieu. Meanwhile, they also secrete anti-inflammatory and pro-regenerative cytokines to promote inflammation resolution as well as tissue repair (Peiseler and Kubes, 2019). Usually, transient activation of kidney recruited immune cells is beneficial for tissue repair and functional recovery because they are helpful in removing the pathogenic factors of kidney injury. However, the accumulation of recruited leukocytes in the renal interstitial compartment promotes chronic inflammation and ultimately leads to renal fibrosis (Gieseck et al., 2018). Emerging evidence has identified altered the trafficking of pathogenic immune cells as crucial drivers of tubulointerstitial inflammation and tissue destruction in the progression of CKD (Schnaper, 2017; Tang and Yiu, 2020). Therefore, the recruited leukocytes might facilitate or undermine the kidney repair process under different conditions. An intriguing issue is which underlying mechanism determines the role of recruited immune cells in the kidney.

## CKD Is an Inflammatory Disease

Chronic inflammation is generally characterized by persistent production of pro-inflammatory cytokines from both circulating and resident effector cells (Anderton et al., 2020). Emerging evidence has demonstrated that systemic chronic inflammation (SCI) is a major pathological event implicated in the development of most chronic diseases or pathological conditions (e.g., chronic heart disease, diabetes mellitus, and CKD; Furman et al., 2017, 2019; Bennett et al., 2018). Under SCI, the low-grade but persistent activation of effector immune cells consistently compromise the normal tissue at the cellular level by direct contact or paracrine of pro-inflammatory cytokines (Kotas and Medzhitov, 2015). Of note, a gradual loss of renal function per se can initiate SCI in disease progression, which is commonly mixed with some other inflammatory conditions, including diabetes mellitus, hypertension, and obesity. For example, DKD is the leading cause of CKD, which has also been considered as an inflammatory disease (Tuttle, 2005). In the condition of DKD, hyperglycemia-induced oxidative stress pathologically activates circulating immune cells, which infiltrate into the involved kidney and aggravate tissue inflammation by abundant production of pro-inflammatory cytokines and chemokines (Donate-Correa et al., 2020). The accumulation of macrophages in the kidney has been correlated to a decline of renal function in DKD patients (Klessens et al., 2017). Furthermore, these infiltrated cells account for the huge release of cytokines, growth factors, reactive oxygen species (ROS), and metalloproteinases, which initiate and amplify the irreversible process of renal fibrogenesis (Matoba et al., 2019). Another common cause of CKD is hypertension that is likewise featured by progressive SCI (Harrison et al., 2011; Chen et al., 2019b). In the progression of hypertension-associated kidney involvements, predominant accumulation of different immune cells, including antigen-presenting cells and T cells, can be detected at the early stage of kidney inflammatory response (Loperena et al., 2018; Norlander et al., 2018). In the pathogenesis, hypertension-associated influence initially activates dendritic cells (DCs) in the kidney largely by promoting the exuberant formation of isoketals. The activated DCs produce abundant cytokines, including interleukin (IL)-6, IL-1β, and IL-23, to recruit T cells from secondary lymphoid organs to the kidney (Kirabo et al., 2014). Meanwhile, hypertension per se can promote T cell infiltration into the kidney by increasing glomerular perfusion pressure (Evans et al., 2017). As a vicious cycle, infiltrated T cells enhance the production of angiotensin (ANG) II and further aggravate hypertension-associated kidney involvements (De Miguel et al., 2010). Collectively, regardless of its pathogenesis, SCI plays a detrimental role in the progression of CKD by promoting renal infiltration of circulating immune cell and aggravating chronic kidney inflammation. It is of clinical significance to further understand the regulatory mechanism of immune cells recruitment in the context of CKD progression.

## Aberrant DNA Methylation Participates in CKD Development

DNA methylation is a common type of epigenetic modification that reversibly affects gene expression without changes in the sequence of nucleotides (Berger et al., 2009; Chen and Riggs, 2011). This process of adding a methyl group to the cytosine is catalyzed by DNA methyltransferases (DNMT), including DNMT1, DNMT3A, and DNMT3B. Generally, DNMT3A and DNMT3B are the major *de novo* DNA methyltransferases, whereas DNMT1 acts as a maintenance enzyme, restoring hemi-methylated DNA to full methylation after replication (Jones and Liang, 2009; Jones, 2012). In the course of cell division, DNA demethylation occurs in the absence of DNMT1 activation. On the other hand, active DNA demethylation can be induced by the mammalian ten-eleven translocation (TET) family, which catalyzes the stepwise oxidation of 5-methylcytosine in DNA to 5-hydroxymethylcytosine (5hmC; Ambrosi et al., 2017). In somatic cells, functional DNA methylation mostly occurs in clusters of CpG dinucleotides (termed CpG islands), and approximately 60–70% of human gene promoters contain a CpG island (Saxonov et al., 2006; Illingworth et al., 2010). DNA methylation is generally believed to induce transcriptional downregulation, either by impairing the interaction between transcription factors and their targets or by recruiting transcriptional repressors with specific affinity for the methylated DNA. At present, known transcriptional repressors can be classified into three families: the methyl-CpG binding domain (MBD) proteins (Hendrich and Bird, 1998; Defossez and Stancheva, 2011), the UHRF proteins (Hashimoto et al., 2008), and the zinc finger proteins (Hudson and Buck-Koehntop, 2018). In brief, DNA methylation, by altering DNA accessibility to gene promoters, induces transcriptional suppression while demethylation is associated with transcriptional activation.

In recent decades, a surge in epigenome-wide association studies (EWAS) has highlighted that DNA methylation can be markedly influenced by environmental exposures, like CKD and SCI (Ligthart et al., 2016; Heintze, 2018). A Renal Insufficiency Cohort (CRIC) identifies enhanced DNA methylation in genes of IQ motif and Sec7 domain 1 (*IQSEC1*), nephronophthisis 4 (*NPHP4*), and transcription factor 3 (*TCF3*) in participants with stable renal function while compared to those with rapid loss of eGFR (Wing et al., 2014). Meanwhile, differential DNA methylation profiles between the two groups can also be detected in the genes associated with oxidative stress and inflammation. Using whole blood DNA, recent EWAS on a large CKD cohort demonstrated that abnormal DNA methylation of 19 CpG sites is significantly associated with CKD development. Importantly, five of these differential methylated sites are also associated with fibrosis in renal biopsies of CKD patients (Chu et al., 2017). The concordant DNA methylation changes can be further identified in the kidney cortex. In animal studies, targeting DNA methylation, either global or gene-specific, can effectively attenuate renal inflammation and fibrosis in progressive CKD (Tampe et al., 2014, 2015; Yin et al., 2017). For example, low-dose hydralazine induces promoter demethylation in the gene of RAS protein activator like 1 (RASAL1), and subsequently attenuates renal fibrosis in the context of AKI to CKD (Tampe et al., 2017). Although hydralazine is an anti-hypertensive medication, the optimum demethylating activity seems to be independent of its blood pressure-lowering effect. Consistently, altered DNA methylation patterns in the renal outer medulla have been shown to induce differential gene expression regulating metabolism and inflammation in the hypertension animal model (Liu et al., 2018), further supporting that DNA methylation is involved in chronic kidney inflammation and a subsequent loss of kidney function. A number of studies have also highlighted the importance of DNA methylation in the pathogenesis of polycystic kidney disease (PKD; Li, 2020). For instance, downregulation of *PKD1* in kidney tissue by hypermethylation may contribute to cyst formation and progression (Woo et al., 2014). Given its relevance to environmental influences, DNA methylation has been intensively explored in DKD. Cumulative evidence suggests that progressive loss of renal function is closely correlated to abnormal DNA methylation in DKD subjects (Swan et al., 2015; Qiu et al., 2018; Gluck et al., 2019; Gu, 2019; Kim and Park, 2019; Park et al., 2019). A recent genome-wide analysis of DNA methylation on 500 DKD subjects reveals that DNA methylation-mediated gene expression likely determines the disease phenotypes, including glycemic control, albuminuria, and kidney function decline. Importantly, further functional annotation analysis indicates that distinct DNA methylation patterns are involved in the pathogenesis of DKD-associated inflammation (Sheng et al., 2020). Collectively, DNA methylation participates in the development of CKD and chronic kidney inflammation in particular.

## DNA Methylation in Peripheral Immune Cells

Chronic kidney inflammation occurs in the process of CKD development regardless of its pathogenesis. Pathologically, it is featured in the cumulative infiltration of diverse immune cells from the circulation into the tubulointerstitial compartment. The recruited immune cells are major participants in the progression of chronic kidney inflammation. Upon infiltration, these cells produce abundant chemokines to establish a pro-inflammatory milieu; meanwhile, they also secrete anti-inflammatory cytokines and pro-regenerative growth factors to promote inflammation resolution as well as tissue fibrosis (Gieseck et al., 2018; Tang and Yiu, 2020). It is of clinical interest to understand the underlying mechanism that regulates the intensity and the time length of the inflammatory response in these circulating immune cells. The current status of epigenetic research acknowledges that altered DNA methylation induces permissive or negative expressions of target genes, which result in pathogenic activation of effector immune cells and the consequential loss of inflammatory homeostasis (Stylianou, 2019). Compelling evidence has revealed that circulating immune cells experience dynamic epigenetic changes in their response to the challenge of either acute insult or chronic pathogenic factors (Keating et al., 2016). The epigenetic “memory” of the previous stimuli can sustain for a long time and affect the future gene expression profile even after their migration from the circulation into the involved kidney. Recent emerging findings support the fact that an aberrant DNA methylation pattern of diverse peripheral immune cells is closely associated with CKD development in multiple disease settings (summarized in Table 1).

**Table 1:** Summary of main changes in DNA methylation in CKD development with immune cells.

Disease	Subjects (References)	Immune cells	Mechanism	Gene(s) modified
DKD	20 Chinese patients with DKD (Chen et al., 2019a)	Peripheral blood mononuclear cell	DNMT1↑	Upstream regulators of mTOR pathway
	181 Pima Indians with diabetes (Qiu et al., 2018)	Blood leukocytes	–	CDGAP, FKBPL, and ATF6B
LN	30 patients with lupus (Zhu et al., 2016)	Peripheral blood mononuclear cell	–	MX1, GPR84, E2F2
	322 women of European descent with lupus, 80 of whom had LN (Mok et al., 2016)	Peripheral blood mononuclear cell, CD4^+^ T cells	–	HIF3A, IFI44, PRR4
	56 patients with lupus (Coit et al., 2015a)	Naïve CD4^+^ T cells	–	IRF7
	SJL mice (Strickland et al., 2015)	CD4^+^T-cells	DNMT1↓	CD70, CD40L, KirL1
	51 patients with lupus (Wardowska et al., 2019)	Dendritic cells	DNMT1↓, MBD2↓	IRFs
	54 female lupus patients (32 patients of European American ancestry and 22 patients of African American ancestry; Coit et al., 2020)	Neutrophils	–	GALNT18
IgAN	30 patients with IgAN (Xia et al., 2020)	Peripheral blood mononuclear cell	DNMT3B↓	C1GALT1
	24 patients with IgAN (Sallustio et al., 2016)	CD4^+^ T cells	–	TRIM27, DUSP3, VTRNA2-1
Hypertensive injury	SS/MCW (JrHsdMcwi) rats, SS/CRL (JrHsdMcwiCrl) rats (Dasinger et al., 2020)	T cells	–	–
CKD/CVD	27 patients with CVD/CKD (Yang et al., 2016)	Monocytes	DNMT1↓	CD40

CKD, chronic kidney disease; LN, lupus nephrites; IgAN, IgA nephropathy; DKD, diabetic kidney disease; CVD, cardiovascular disease; DNMT, DNA methyltransferase; mTOR, mammalian target of rapamycin; CDGAP, Cdc42 GTPase-activating protein; FKBPL, FK506-binding protein-like; ATF6B, activating Transcription Factor 6 Beta; MX1, myxovirus resistant 1; GPR84, G protein-coupled receptor 84; E2F2, E2F transcription factor 2; HIF3A, hypoxia-inducible factor 3α gene; IFI44, interferon induced protein 44; PRR4, proline-rich protein 4; IRF, interferon regulatory factor; KirL; GALNT18 polypeptide nacetylgalactosaminyltransferase 18; C1GALT1, core 1 synthase, glycoprotein-N-acetylgalactosamine 3-beta-galactosyltransferase 1; TRIM27, tripartite motif-containing 27; DUSP3, dual-specificity phosphatase 3; VTRNA2-1, vault RNA 2-1.

Firstly, we have recently reported that chronic hyperglycemia induces over-expression of DNMT1 and subsequent aberrant DNA methylation of multiple regulator genes of the mechanistic target of rapamycin (mTOR) in peripheral blood mononuclear cells (PBMCs). These effector cells in turn activate and migrate into the involved kidney with the abundant secretion of inflammatory cytokines, resulting in persistent kidney inflammatory injuries and progressive fibrosis (Chen et al., 2019a). By adoptive transfer, we confirm that circulating PBMCs with “inflammatory memory” can aggravate DKD progression in the recipient animals. Of clinical importance, we demonstrate that the inhibition of DNA methylation by targeting DNMT1 promotes the regulatory phenotype of circulating immune cells and improves the diabetic inflammatory state and the long-term outcome of DKD. Aberrant DNA methylation is also observed in PBMCs from lupus nephritis (LN) patients. Hypomethylated CpG sites can be detected in the promoter region of interferon (IFN)- and toll-like receptor (TLR)-related genes, which are highly associated with the pathogenic inflammatory condition of LN progression (Mok et al., 2016; Zhu et al., 2016). These findings highly support the fact that the differential methylation of genes regulating the inflammatory activity of PBMCs has a causal role in the pathogenesis of LN. In addition, we have observed that mRNA expression of *DNMT3B* is notably increased in PBMCs isolated from immunoglobulin A nephropathy (IgAN) patients (Xia et al., 2020).

**Figure 1:**
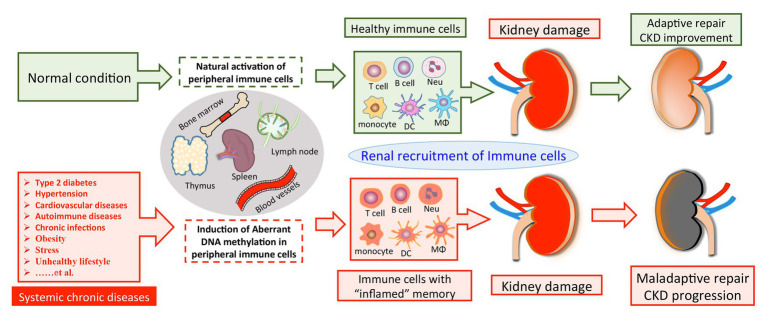
A model of DNA methylation in peripheral immune cells in the pathogenesis of CKD development. Chronic pathogenic conditions induce aberrant DNA methylation in peripheral immune cells, leading to enhanced activities of pro-inflammatory genes and a cell-type switch toward “inflamed” effectors. Normally, renal recruitment of circulating immune cells can facilitate adaptive repair and improve the outcome of kidney damage. On the other hand, peripheral immune cells with “inflamed” DNA methylation profile may constantly migrate into the diseased kidney and overact tissue inflammation, which consequentially results in maladaptive repair and CKD progression. CKD, chronic kidney disease; Neu, neutrophil; DC, dendritic cell; Mϕ, macrophage.

Based on these findings, we propose that SCI occurs and progresses in the condition of CKD derived from multiple primary and secondary diseases, such as hyperglycemia, hypertension, autoimmune disorder, and chronic infection. These chronic stimuli substantially alter the DNA methylation profile of circulating immune cells, leading to enhanced activities of pro-inflammatory genes and a cell-type switch toward inflammatory effectors. The altered DNA methylation might act as “epigenetic memory” and sustain in circulating immune cells for a long time. It thereby pathologically and persistently activates the inflammatory response of immune cells, which continue to participate in chronic tissue injury after their kidney recruitments. It might partly explain why a subset of CKD is characterized by ongoing kidney inflammation and irreversibly progresses to ESRD even when treatment targets have been achieved, like glycemic recovery and blood pressure control (Figure 1). Of note, leukocytes are composed of a variety of circulating immune cells and DNA methylation affects genes transcription activity by a cell type-specific manner. Although emerging evidence has revealed abnormal DNA methylation in both B cells (Absher et al., 2013; Fali et al., 2014; Scharer et al., 2019; Breitbach et al., 2020; Wardowska, 2020) and neutrophils (Lande et al., 2011; Coit et al., 2015b, 2020) in the condition of SLE, there is a lack of data derived from studies with kidney involvements by far. Therefore, we next discuss the potential role of DNA methylation in CKD development with a focus on T cell and monocyte lineages.

## DNA Methylation in T Cell Lineages

Upon antigen stimulation, naïve T cells differentiate into several lineages, including T helper (Th)1, Th2, Th17, and regulatory T (Treg) cells. Th1 cells control intracellular bacterial infection, while Th2 cells initiate antibody response against the extracellular pathogens. During the polarization of CD4^+^ T cells toward Th1, DNA hypomethylation occurs in Th1 cytokine genes (such as interferon gamma, IFNγ) whereas Th2 cytokine genes achieve DNA methylation, and vice versa in the polarization of Th2 cells. Evidence showed that the imbalance of Th1/Th2 cytokine profiles play a crucial role in the pathogenesis of IgAN (Suzuki and Suzuki, 2018). In the early stage of IgAN studied in ddY mice, strong polarization toward Th1 can be observed (Suzuki et al., 2007). A genome-wide screening for DNA methylation shows that the ratio of IL-2 to IL-5 is significantly elevated, indicating a Th1 shift of CD4^+^ T cells in IgAN (Sallustio et al., 2016). In brief, this Th1/Th2 polarization is associated with three specific aberrantly methylated DNA regions in peripheral CD4^+^T cells from IgAN patients. Low methylation levels are observed in genes involved in T cell receptor (TCR) signaling, including tripartite motif-containing 27 (*TRIM27*) and dual-specificity phosphatase 3 (*DUSP3*). Meanwhile, a hypermethylated region can be detected in the miR-886 precursor and is associated with decreased CD4^+^ T cell proliferation following TCR stimulation. Therefore, aberrant DNA methylation causes reduced TCR signal strength and the low activation of CD4^+^ T cells in the pathogenesis of IgAN.

Th17 cells are characterized by the signature production of cytokines such as IL-17A and IL-17F and the expression of the key transcription factor retinoic orphan receptor γt (RORγt; Cua et al., 2003). Due to their pro-inflammatory phenotype, Th17 cells are capable of protecting against infections on mucosal surfaces (Park et al., 2005) but contribute to the development of renal inflammatory diseases (Turner et al., 2010). On the other hand, Treg cells are characterized by the expression of forkhead box P3 (*Foxp3*) and the production of anti-inflammatory cytokines (e.g., IL-10 and transforming growth factor-β; Lu et al., 2017) and usually have a pivotal role in dampening chronic kidney inflammation (Chen et al., 2016; Sharma and Kinsey, 2018). Changes in epigenetic status at the *Foxp3* and *IL17* gene loci are essential for the polarization of CD4^+^ T cells toward the Treg or Th17 cells (Yang et al., 2015; Lu et al., 2016). Peripheral CD4^+^ T cells from SLE patients were presented with decreased expression of regulatory factor X 1 (*RFX1*), which causes DNA demethylation in the IL17A locus of CD4^+^ T cells and thereby promotes Th17 cell differentiation (Zhao et al., 2018). On the other hand, abnormal epigenetic regulation of *Foxp3* in Treg cells has been documented in SLE patients, which suggests that hypermethylation of the Foxp3^+^ promoter region is associated with a decreased proportion of Treg cells and increased disease activity (Zhao et al., 2012). Of clinical significance, DNA methylation levels of the *Foxp3* promoter region can be markedly suppressed by effective treatment, which consequently downregulates Foxp3 expression and promotes CD4^+^CD25^+^ Treg cells.

In addition, recent EWAS has revealed that unique DNA methylation patterns in CD4^+^ T cells are closely related to disease activity. In SLE, the DNA methylation state in peripheral naïve CD4^+^ T cells is significantly different between patients with and without renal involvement (Coit et al., 2015a). Increased DNA methylation in multiple IFN-regulated genes is closely associated with the onset of LN. Moreover, a lupus susceptibility gene, the type-I interferon master regulator gene (*IRF7*), is specifically demethylated as shown in patients with LN. Consistently, the modification of DNA methylation, by targeting DNMT1 expression in CD4^+^ T cells, contributes to the development of LN-like glomerulonephritis in animals (Strickland et al., 2015).

As described above, abnormal epigenetics is implicated in the pathogenesis of hypertensive renal injury due to its influence on immune homeostasis. It is known that high salt intake is the major cause of hypertension and intriguingly associated with obesity, independent of energy intake (Ma et al., 2015). An intriguing question is whether and how environmental influences, like unhealthy diet, might induce aberrant epigenetic changes in immune cells that subsequently participate in hypertension-associated kidney inflammatory involvement. The Dahl salt-sensitive (SS) rat is a genetic model of hypertension and renal disease that is accompanied with immune cell activation in response to a high-salt diet (Mattson et al., 2006). In SS rats, a high-salt diet induced increasing global methylation rate in circulating and kidney T cells, of which differentially methylated regions (DMRs) are more prominent in animals with a pronounced hypertensive phenotype. Importantly, the application of decitabine, a hypomethylating agent, significantly attenuates hypertension and renal inflammatory injury in SS rats (Dasinger et al., 2020). In-depth RNA-seq analysis on kidney T cells has revealed the upregulation of multiple inflammatory and oxidative genes in response to a high-salt diet, which are inversely correlated with DNA methylation levels. These genes are known to play an important role in the development of salt sensitivity in the SS rat (Zheleznova et al., 2016). Collectively, these findings thereby highlight the important role of DNA methylation in linking the influence of abnormal environment/diet to the clinical manifestations of hypertension-associated involvements, which might be at least partly mediated by pathologically activated T cells.

## DNA Methylation in Monocyte Lineages

Monocytes, representing the mononuclear phagocyte system, are the largest type of circulating immune cells and can differentiate into macrophages (Mϕ) and myeloid lineage dendritic cells (DCs). Multiple lines of evidence have confirmed the fundamental roles of monocyte lineage in the inflammatory progression of CKD (Heine et al., 2012; Kinsey, 2014; Bowe et al., 2017). Generally, Mϕ can be divided into two subsets, classically activated Mϕ (M1) and alternatively activated Mϕ (M2), depending on their activation paradigm and cellular functions. The classic M1 macrophages commonly produce pro-inflammatory cytokines and cytotoxic mediators contributing to acute and chronic tissue inflammation. On the other hand, M2 macrophages are mostly implicated in inflammation resolution, tissue remodeling, and fibrogenesis by secreting various anti-inflammatory cytokines, growth factors, and proangiogenic cytokines (Wang et al., 2014; Tian and Chen, 2015). In the context of DKD, Mϕ constitutes a major part of infiltrated leucocytes and their accumulation is associated with the progression of diabetic status and renal pathological changes (Chow et al., 2004; Tesch, 2010). Importantly, M1/M2 ratio is positively associated with the progression of chronic inflammation into pathogenic fibrosis during CKD development (Tang et al., 2019; Zhang et al., 2019). Recent studies have revealed an essential role of epigenetic regulation in the phenotype switch of M1 and M2. For example, DNMT3B plays an important role in regulating macrophage polarization and is expressed relatively less in M2 compared to M1 (Yang et al., 2014). Deletion/inhibition of DNMT1, either pharmacologically or genetically, contributes to M2 alternative activation in obesity (Wang et al., 2016), which is known as a typical type of SCI. Under the pathological conditions of hyperlipidemia and type 2 diabetes mellitus, DNA methylation alterations steer the Mϕ phenotype toward pro-inflammatory M1 as opposed to the tissue repairing M2 phenotype by differentially methylating gene promoters of M1 and M2 (Babu et al., 2015).

Besides differentiation into Mϕ, monocytes can be classified into various subsets with diverse inflammatory phenotypes based on their cell surface markers expression (Zawada et al., 2012), which similarly can be interfered with in the stage of CKD. Accumulation of uremic toxins during CKD progression induces aberrant DNA methylation that affects some transcription regulators that are important for monocyte differentiation (Zawada et al., 2016). Similar to other chronic diseases, CKD can promote a pro-inflammatory phenotype of monocytes *via* the DNA hypomethylation of CD40, which activates and contributes to inflammatory involvements and disease progression (Yang et al., 2016). DCs can be generally divided into two groups, myeloid dendritic cells (mDCs) and plasmacytoid dendritic cells (pDCs; Kitching and Ooi, 2018). Although the majority of DCs within the kidney are cDCs, active pDCs can migrate and contribute to tissue inflammation in nephritic kidneys (Fiore et al., 2008; Tucci et al., 2008). Myeloid DCs (mDCs, BDCA1^+^ or BDCA3^+^ DCs) are also shown to increase in the renal tubulointerstitium of patients with LN (Fiore et al., 2008). DNA methylome of peripheral DCs reveals that global DNA hypermethylation in LN patients is associated with severe kidney involvement (Wardowska et al., 2019). Taken together, current evidence supports the fact that aberrant DNA methylation induces an inflammatory switch of monocyte lineage, which contributes to the development of chronic kidney inflammation in multiple chronic disease settings, like obesity, hypertension, diabetes, lupus, and CKD.

## Summary and Perspectives

**Figure 2:**
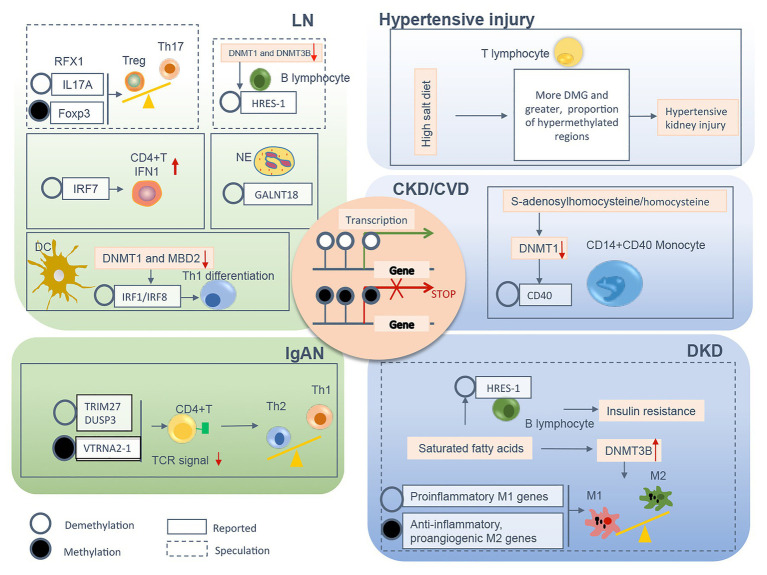
The relevant DNA methylation profiles in immune cells from CKD patients are summarized by different chronic pathogenic conditions, including LN, IgAN, hypertensive kidney injury, DKD, and uremia. Demethylation or methylation of certain genes regulates immune cell phenotype shift/differentiation, or pro/anti-inflammation signal, therefore contributes to uncontrolled kidney inflammation and CKD progression. The mechanism boxed off with solid lines is documented in CKD with different etiology, whereas the one with dashed lines is speculated to relate to the development of kidney diseases founded on circumstantial evidence. CKD, chronic kidney disease; LN, lupus nephrites; IgAN, IgA nephropathy; DKD, diabetic kidney disease; CVD, cardiovascular disease; Treg, regulatory T; Th, T helper; NE, neutrophil; DC, dendritic cell; TCR, T cell receptor; M1, classically activated macrophage; M2, alternatively activated macrophage; IFN, interferon; Foxp3, forkhead box P3; DNMT, DNA methyltransferase; MBD, methyl-CpG binding domain; RFX, regulatory factor X; HRES-1, human T cell lymphotropic virus-related endogenous sequence-1; IRF, interferon regulatory factor; GALNT18, polypeptide nacetylgalactosaminyltransferase 18; TRIM27, tripartite motif-containing 27; DUSP3, dual-specificity phosphatase 3; VTRNA2-1, vault RNA 2-1.

In summary, a variety of pathological conditions induce an aberrant DNA methylation profile in circulating immune cells with a cell-type specific manner, leading to a phenotype switch toward the inflammatory side (Figure 2). These “inflamed” immune cells sustain enhanced inflammatory activity upon the recruitment into diseased kidneys and consequentially participate in chronic kidney inflammation and CKD progression. DNA methylation-targeted treatment by either inhibiting methylation (e.g., 5-azacytidine) or activating demethylation (e.g., hydralazine) have been explored to ameliorate kidney injury in several preclinical studies (Table 2), though some of the interventions have nephrotoxic potential in the clinical setting. A series of novel therapeutic methods, such as modified oligonucleotide inhibitors and small RNA molecules targeting DNMTs, have yet to be tested in the setting of kidney disease (Xu et al., 2016). Meanwhile, there is a lack of intervention strategies specifically targeting immune cells. Given its complex roles in cell biology, clinicians should comprehensively assess the therapeutic value, as well as the potential risk of targeting DNA methylation in immune cells. An in-depth understanding of DNMTs functions in different scenarios might help to develop effective strategies to restore immune homeostasis with consideration of the timing, the signaling intensity, and the disease settings. In future mechanistic research, it remains necessary to clarify the causal relationship between DNA methylation and CKD development, since it is technically hard to separate “driver” events from “passenger” events in the setting of SCI. A combined application of current cutting-edge technologies, like single-cell epigenomic methods of ATAC-seq (Mezger et al., 2018) and single-cell RNA-seq (Kolodziejczyk et al., 2015), may be able to provide a solution to this problem.

**Table 2:** Summary of existing potential treatment of CKD targeting on DNA methylation.

Drugs	Target	Model	Effect	References
5-azacytidine	DNMT inhibitor	Mouse folic-acid-induced AKI	Fibrosis↓	Bechtel et al., 2010
		Mouse db/db DKD	Renal function↑, proteinuria↓, Renal injury↓	Zhang et al., 2017
Hydralazin	Demethylating activity: induction of TET3	Mouse UUO	Fibrosis↓	Tampe et al., 2015
		Mouse IRI, Mouse hRASAL1-pTreTight transgenic	*RASAL1* promoter demethylation↑, Fibrosis↓, Renal function↑	Tampe et al., 2017
BMP7	Normalization of aberrant Rasal1 methylation, which is dependent on Tet3-mediated hydroxymethylation	Mouse streptozotocin -induced DKD, mouse UUO, *COL4A3*-deficient Alport mice, mouse 5/6 nephrectomy-induced CKD	Fibrosis↓	Tampe et al., 2014
Decitabine	DNMT inhibitor	Mouse UUO	Fibrosis↓	Bechtel et al., 2010
5'-deoxy-5'-methylthioadenosine	Indirect inhibitor of methyltransferases	Mouse MRL/lpr lupus	IgG deposition and cellular infiltration in the kidney↓	Yang et al., 2013

DNMT, DNA methyltransferase; AKI, acute kidney injury; DKD, diabetic kidney disease; TET, ten-eleven translocation; UUO, unilateral ureteral obstruction; IRI, ischemia-reperfusion injury; RASAL1, RAS protein activator like 1; BMP7, bone morphogenetic protein 7.

## Author Contributions

GC conceived the review. X-JC and HZ collected literature data, interpreted literature, and wrote the manuscript. FY and YL created and revised the figures and tables. GC oversaw the work and revised the manuscript. All authors contributed to the article and approved the submitted version.

### Conflict of Interest

The authors declare that the research was conducted in the absence of any commercial or financial relationships that could be construed as a potential conflict of interest.
